# IS THE ANATOMICAL SEQUENCE OF GASTRIC AND BILIARY ANASTOMOSIS IN THE PANCREATODUODENECTOMY RECONSTRUCTION THE CAUSE OF AN INCREASE IN THE INCIDENCE OF CHOLANGITIS? A TECHNICAL VARIANT PRESENTATION AND INITIAL RESULTS

**DOI:** 10.1590/0102-672020220002e1688

**Published:** 2022-09-16

**Authors:** Gustavo Adrian NARI, Alesio LOPEZ, Jose Luis LAYUN, Daniela MARIOT, Flavia LOPEZ, Maria Eugenia DE-ELIAS

**Affiliations:** 1 Transito Caceres de Allende Hospital, Surgery Unit - Córdoba, Argentina;; 2 La Canada Sanatorium, Oncology Surgery Unit - Córdoba, Argentina.

**Keywords:** Pancreatoduodenectomy, Postoperative Complications, Cholangitis, Anastomosis, Surgical, Pancreaticoduodenectomia, Complicações Pós-Operatórias, Colangite, Anastomose Cirúrgica

## Abstract

Several methods have been proposed for the reconstruction of digestive transit after pancreatoduodenectomy. Biliary anastomosis positioned before gastric anastomosis helps reduce postoperative reflux and cholangitis.

**AIMS:**

The objective of this study was to present the anatomical sequence of gastric and biliary continuity after pancreatoduodenectomy in patients with pancreatic tumor and to evaluate the short- and long-term results in an initial series of cases.

**METHODS:**

Two techniques were used: one with Roux-en-Y reconstruction and pancreaticojejunostomy and the other with a single jejunal loop and pancreatogastroanastomosis. In both the cases, the gastric anastomosis was placed performed before the biliary one. An analysis of demographic data, Wirsung’s duct and common bile duct dilatation, the use of percutaneous drainage, and postoperative complications was carried out.

**RESULTS:**

A total of seven patients (four men and three women), with a mean age of 62 years, underwent surgery. All cases had Wirsung’s duct and common bile duct dilatation. A percutaneous external biliary drainage was performed in four patients. There were three postoperative complications: one related to delayed gastric emptying and two related to wound infections. During a median follow-up of 12 months, no episode of cholangitis was recorded.

**CONCLUSIONS:**

Elevated percentages of cholangitis are reported in different reconstructions after pancreatoduodenectomy, and it is difficult to conclude reflux as the main etiology. The proposed gastric and biliary reconstructions show conforming results, facilitating posterior endoscopic access. Late follow-up and large number of cases may help assess whether the etiology of postoperative cholangitis is reflux or other factors unrelated to the order of the anastomoses.

## RESUMO - RACIONAL

Múltiplas são as propostas de reconstrução do trânsito digestivo após as pancreadoduodenectomias. A anastomoses biliar posicionada antes da anastomose gástrica oferece argumentos de evitar refluxo e colangite pós-operatória.

**OBJETIVOS:**

apresentar a técnica de continuidade gástrica e biliar com sequência anatômica após pancreatoduodenectomia em portadores de adenocarcinoma de pâncreas e avaliar os resultados em uma série inicial de casos.

**MÉTODOS:**

Foram utilizadas duas técnicas, uma com reconstrução em Y de Roux e pancreaticojejunostomia e outra com alça única de jejuno e pancreatogastroanastomose. Em ambos, a anastomose gástrica foi colocada antes da biliar. E análise de dados demográficos, dilatação do ducto de Wirsung e ducto biliar comum, uso de drenagem percutânea e complicações pós-operatórias.

**RESULTADOS:**

Foram operados 7 doentes: 4 homens e 3 mulheres, com média de idade de 62 anos. Todos os casos apresentavam dilatação do ducto de Wirsung e ducto biliar comum. Em 4 dos casos foi realizada drenagem biliar externa percutânea. Ocorreram 3 complicações pós-operatórias, 1 esvaziamento gástrico retardado e 2 infecções de ferida operatória. Durante o acompanhamento médio de 12 meses, não foram registrados episódios de colangite.

**CONCLUSÕES:**

Porcentagens elevadas de colangite são relatadas nas diferentes reconstruções após pancreatodudenectomias, sendo difícil atribuir de forma absoluta o refluxo como a principal etiologia. As reconstruções gástrica e biliar propostas são mais harmoniosas, além de facilitar o acesso endoscópico posterior. Seguimento tardio e número maior de casos, pode esclarecer se a etiologia da colangite pós-operatória pode ser o refluxo ou a outros fatores não relacionados à ordem das anastomoses.

## INTRODUCTION

Researchers like Codevilla (1878), Kausch (1912), Whipple (1938 and 1944), Cattell (1943), and Child (1944), to name a few, have proposed techniques for the reconstruction of different continuities after pancreatoduodenectomy (PD, with an intention to reduce complications such as the number of postoperative pancreatic fistula, delayed gastric evacuation, and hemorrhage with such modifications. These proposed techniques include a single jejunal loop (with or without Braun anastomosis), Roux-en-Y with one loop as alimentary anastomosis and one loop as biliary and pancreatic anastomosis, Roux-en-Y with one loop as pancreatic anastomosis and one loop as biliary and gastric, pancreaticogastric anastomosis, pylorus preserve PD, and the elevation of the jejunal loop in a transmesocolic way^2^ ^,^ ^7^ ^,^ ^8^ ^,^ ^15^ ^,^ ^23^ ^,^ ^24^.

Few studies have focused purely and exclusively on biliary complications and postoperative cholangitis^6^ ^,^ ^10^ ^,^ ^11^ ^,^ ^13^ ^,^ ^17^. One common element of all the techniques described is: the sequence of the biliary and gastric anastomosis are reversed to the anatomical or normal sequence (stomach first, bile duct later) such that food reflux within the bile duct and subsequent cholangitis as a consequence of the absence of the papilla of Vater are reduced. Another effect associated with this change in sequence is a distance of no less than 40-60 cm between the biliary and gastric anastomosis^11^. In the same way, the jejunal loop should be placed isoperistaltically and the biliary anastomosis in the first jejunal loop, which would result in greater mobility.

Research has found that choledocoduodenostomy (CD) may provide more episodes of cholangitis when compared with hepaticojejunostomy (HJ). On the other hand, hepaticoduodenostomy (HD) (in which there is an anatomical sequence) in the treatment of cysts of the common bile duct has not shown a high percentage of cholangitis in the short- and long-term follow-up^16^.

The aim of this study was to present anatomical sequence of the gastric and biliary continuity after PD and to assess the short- and long-term results of an initial series.

## METHODS

### Surgical Technique

Once the duodenum-pancreas was resected, the continuity of the digestive transit was reconstructed in two ways:

Type I . *Reconstruction on a Roux-en-Y loop*: in one loop, an end-to-end pancreatic-jejunum anastomosis was performed with Hunt stitches^14^, and in the other loop, an end-to-end gastroenterostomy was performed, followed by an end-to-side hepatic-jejunum anastomosis, which is placed approximately 15 cm away.

Type II . *A Child type loop reconstruction:* an end-to-end anastomosis was performed between the jejunal loop and the stomach, followed by an end-to-side biliary anastomosis, which is placed approximately 15 cm away.

**Figure 1 -:**
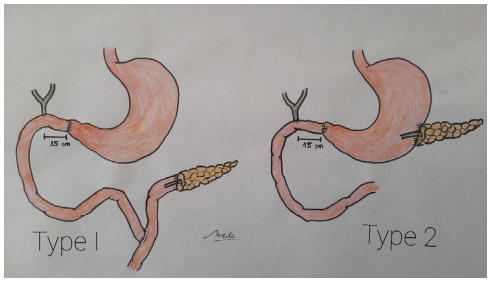
Anatomical sequence of the gastric and biliary anastomosis. Type I: gastric and biliary anastomosis (anatomical sequence) on one of the branches of a Roux-en-Y, in the other branch an end-to-end pancreatojejunoanastomosis. Type II: gastric and biliary anastomosis (anatomical sequence) on a jejunal loop with pancreatic-gastric anastomosis.

Pancreatic continuity was established through a pancreatogastroanastomosis. In both types of reconstruction, pyloric preservation was performed^2^ (Figure 1).In all the patients, the anastomosis between the stomach and the jejunum was performed through a two-layer running suture with absorbable thread. In the bilioenteric anastomosis, the posterior plane was made with separate stitches that were tied in a deferred manner, while the anterior face was made through a running suture with absorbable thread. No patient had the biliary anastomosis intubated. A catheter was placed in the duct of Wirsung to direct the pancreatic secretion toward the used organ. In the Child type reconstruction, the jejunal loop was raised behind the mesenteric vessels, reconstructing the duodenal “C,” while in the one using a Roux-en-Y, it was the loop that communicates with the pancreas that was raised in this way, while the alimentary loop was transmesocolic ascended. A nasogastric tube was set up and inserted until the biliary anastomosis passed and served to initiate early feeding. Two multilumen drains were placed in the abdominal cavity: one under the pancreatic anastomosis and the other in the foramen of Winslow.

We retrospectively analyzed data collected prospectively from patients undergoing PD in the past 2 years using the anatomical sequence of the gastric and biliary anastomosis.

Demographic data of the patients, laboratory values, diameter of the bile and pancreatic duct, and the use of preoperative biliary drainage and neoadjuvant chemoradiotherapy were analyzed.

The bile duct was considered dilated if its diameter is ≥8 mm, and the duct of Wirsung dilated when it measured >3 mm. Data of the surgery, hospital stay, complications, and postoperative follow-up were also analyzed. For postoperative pancreatic fistula, delayed gastric evacuation, and bleeding, the ISGPS classification was employed^1^ ^,^ ^21^ ^,^ ^22^, and for biliary fistula, the ISGLS classification was employed^3^.

Cholangitis was classified according to the 2013 Guidelines of Tokyo as follows^12^: early when diagnosed in the first 30 postoperative days, late when diagnosed after postoperative day 30, and refractory when repeated three times or more.

Complications were scored according to the Dindo-Clavien classification^5^. The amylase dosage from the multilumen drainage tube placed in the pancreatic anastomosis was measured on postoperative days 1, 3, and 5, sometimes on day 7.

The postoperative follow-up was carried out in the Oncology Unit, with a computed tomography, clinical analysis, and tumor markers when necessary. Due to the small number of cases, the quantitative variables were evaluated with the range, median, and standard deviation, while the qualitative variables with the average percentage. All patients gave informed consent.

## RESULTS

From January 2019 to January 2021, a total of 26 pancreatic resections were performed in the Surgical Unit, of which 5 were distal resections and 21 were PDs. Figure 1 shows seven cases with different reconstruction techniques. Table 1 shows demographic and clinical data of the patients. Four patients had adenocarcinoma of the pancreas head, one a primary lymphoma, and two patients had papilla tumors. All patients presented dilatation of the main bile duct and the duct of Wirsung.

**Table 1 -:** Demographic and clinical data.

Variable	n	range	Mean SD
Male sex	4 (57.1%)		
Age	62 (years)	52-65	(4.1)
BMI	22 (kg/m^2^)	19-26	(2)
Total preoperative bilirubin	13 (mg/dL)	4-17	(4.7)
Preoperative alkaline phosphatase	612 (IU/L)	415-815	(147.9)
Preoperative albumin	3.5 (g/dL)	3.1-4	(0.26)
Bile duct diameter by ultrasound	14 (mm)	11-18	(2.6)
Wirsung dilation by tomography	7 (100%)		
Percutaneous biliary drainage placement	4 (57.1%)		
Pancreatic adenocarcinoma	4		
Papilla tumor	2		
Primary pancreatic lymphoma	1		
Preoperative chemoradiotherapy	1 (14.2%)		

N: number; SD: Standard deviation; BMI: Body Mass Index.

Percutaneous preoperative drains were placed in the bile duct in four patients. In other four patients, the biliary drainage was external and it was always positioned in the intrahepatic bile duct (Table 1).

The pancreas was found to be increased in consistency in 85.7% of the cases. The reconstruction was Type I in five cases and Type II in two cases. The postoperative complications were found in 3 (42.8%) cases, with a delay in gastric emptying being the most serious complication, which was managed expectantly with nasogastric intubation, and the remaining two were surgical wound infections. The average length of hospital stay was 7 days, and an ERAS protocol was applied in four patients. Two patients with adenocarcinoma had recurrence at the lymphatic level detected in PET scan. The mean follow-up was 12 months (Table 2)*.*

**Table 2 -:** Data regarding surgery and follow-up.

Variable	n	range	Mean SD
Hard pancreatic tissue	6 (85.7%)		
Type I - Roux-en-Y PP:	2 cases		
Type II - Child with PP Reconstruction technique:	5 cases		
Complications	3 (42.8%) (Dindo-Clavien: Type I: 2 and Type II: 1)		
Pancreatic fistula	No		
Gastric evacuation delay	1 - Grade B		
Hemorrhage	No		
Transient jaundice	No		
Cholangitis	No		
Surgical site infection	2		
Operative time in minutes	320	190 - 360	(50.8)
Transfusion of blood products	1 (14.2%)		
Hospital stay in days	7	6-17	(3,6)
Follow-up in months	12	3-19	(4,8)
Recurrence	2 (28.5%)		

N: number; SD: Standard deviation;PP: pyloric preservation.

**Figure 2 -:**
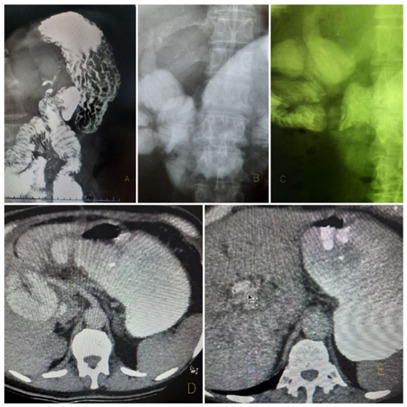
Postoperative radiological and tomographic findings.

In the late follow-up, four patients presented some degree of pneumobilia observed by tomography, of which one patient had a slight but persistent elevation of alkaline phosphatase which does not reach twice the normal value. None of the patients had clinical or laboratory signs suggestive of cholangitis or transient elevation of bilirubin. In addition to tomography, a serial esophagus-gastro-duodenal radiograph was performed in three patients in order to observe the amount of contrast reflux through the biliary anastomosis. Figure 2 shows light staining of the bile duct in three serial esophageal-gastro-duodenal radiographs after the contrast has filled the stomach and a large part of the jejunum. The same finding can be observed in the axial section of the tomography, with the rise of contrast within the bile duct.

## DISCUSSION

Reconstruction of biliary and gastric transit after PD is performed, leaving a distance of >40 cm between one and the other. The motive for performing these anastomoses, with an inverted sequence, is that this reduces the episodes of cholangitis due to reflux of the food content within the biliary anastomosis that would occur if the sequence were anatomical (gastric first and biliary later). It is probable that the origin of these concepts is the number of previously reported episodes of cholangitis in CD, which according to some authors was around 10%^11^. Suiffet et al. in a review of 2146 patients undergoing CD reported an incidence of 0.73% (16 patients)^19^. The presence of the blind sac in between the anastomotic mouth and the papilla could justify stagnation and subsequent ascending infection.

Patil et al.^16^ in a review of 56 patients used HD as a biliary-digestive anastomosis in the treatment of common bile duct cysts and reported a single cholangitis secondary to anastomotic stenosis 18 years after its preparation (0.56%), which would be motive of doubt on the exaggerated reflux within the bile duct as the primary cause of cholangitis in this type of anastomosis. These authors performed the anastomosis with the duodenum 2 cm from the pylorus and always at the level of the biliary confluence, although they did not report its diameter.

There is little literature that refers to the biliary complications of PD. Cholangitis incidence is reported to be between 1 and 18.6%^2^ ^,^ ^6^ ^,^ ^8^ ^,^ ^9^ ^,^ ^11^ ^,^ ^13^ ^,^ ^20^ ^,^ ^23^ ^,^ ^24^, with most episodes appearing within the first 30 days^8^ ^,^ ^13^. Ueda et al.^20^ found 17 of the 18 patients with refractory cholangitis reporting the infection in the first year after surgery.

There is a great variability in the percentage of cholangitis reported in both CD and post-PD biliary anastomoses, as well as a low percentage in HD, thus making it difficult to confirm whether reflux is the main cause of episodes of cholangitis in those anastomoses where the bile duct is anastomosed below the stomach.

HD is the procedure that most closely resembles the reconstruction of the anatomical sequence, which we have used in our patients. Comparing HJ and HD in children in the treatment of common bile duct cysts, Santore et al. concluded that that in the follow-up, patients who underwent HJ had more cholangitis than those who underwent HD (15 vs. 3%)^18^. In the same way, but comparing both anastomoses in liver transplantation, the percentage of cholangitis was practically the same between both (HD 14 vs. HJ 12%)^4^.

Some causes of cholangitis in PD include anastomotic stenosis, calculi, intestinal obstruction, afferent loop syndrome, and jejunal peristalsis disorders^11^ ^,^ ^13^. The cause of the early episodes of cholangitis, which are the most frequent^8^, could be attributed to minimal biliary stricture due to acute inflammation, ileus, peristalsis disorders, and contamination by resistant germs^13^, while the late episodes would be associated with a stenosis of the anastomosis. Duconseil et al.^6^ have found that the main predictor of stenosis is a thin bile duct. Other authors^9^ reaffirm this concept emphasizing that a bile duct smaller than 15 mm in diameter is a risk factor for stenosis and also proposed the performance of a hepaticoplasty to increase the diameter of the anastomotic mouth and to increase bile flow into the intestine. Our patients had a mean diameter of 14 mm. and although there were no episodes of cholangitis in the average 12-month follow-up, the vast majority of stenoses occur in the first 2 postoperative years; therefore, we consider that the time monitoring is insufficient. Other factors associated with cholangitis in the postoperative period are resection for benign pathology, prolonged surgery time, and persistent elevation of alkaline phosphatase^20^, the latter with a value higher than 440 will lead to stenosis^11^. These same authors and others also suggested that the use of preoperative biliary drainage or stents would foment the appearance of cholangitis, causing micro-trauma in the bile duct^10^ ^,^ ^11^. Because we agree on this last observation, we leave the percutaneous drains, preferably before biliary confluence, working as external biliary drainage. We have noticed that in those patients with drains that run through the common bile duct, they produce a traumatic choledochitis that, depending on the time it is left at the site, makes its dissection difficult during surgery and requires an anastomosis to be made over an inflamed biliary border. This anatomical sequence was employed in seven patients without episodes of cholangitis, and this new arrangement was more harmonious, favoring endoscopic access in a natural way to the bile duct and reducing the time of preparation by avoiding an anastomosis, more than when Roux-en-Y was used. In the imaging tests, we were able to observe minor reflux with complete filling of the stomach and a significant portion of the jejunum in patients who underwent serial radiography.

## CONCLUSION

This study includes few patients and a short-term follow-up time, which limited our findings. Future studies with a greater number of cases are suggested so that we can assess whether the cause of cholangitis is reflux or other factors not related to the order of the anastomoses.
